# Development and application of a multi-modal task analysis to support intelligent tutoring of complex skills

**DOI:** 10.1186/s40594-018-0108-5

**Published:** 2018-04-15

**Authors:** Anna Skinner, David Diller, Rohit Kumar, Jan Cannon-Bowers, Roger Smith, Alyssa Tanaka, Danielle Julian, Ray Perez

**Affiliations:** 1 Black Moon, LLC, Washington, DC USA; 2 Raytheon BBN Technologies, Cambridge, USA; 3 Cannon-Bowers Consulting, Orlando, USA; 4 Florida Hospital Nicholson Center, Celebration, USA; 5 Office of Naval Research, Arlington, USA

**Keywords:** Task models, Task analysis, Intelligent tutoring system, Robot-assisted surgery, Psychomotor skills, Cognitive skills, Perceptual skills, Multi-modal task, Simulation-based training

## Abstract

**Background:**

Contemporary work in the design and development of intelligent training systems employs task analysis (TA) methods for gathering knowledge that is subsequently encoded into task models. These task models form the basis of intelligent interpretation of student performance within education and training systems. Also referred to as expert models, they represent the optimal way(s) of performing a training task. Within Intelligent Tutoring Systems (ITSs), real-time comparison of trainee task performance against the task model drives automated assessment and interactive support (such as immediate feedback) functionality. However, previous task analysis (TA) methods, including various forms of cognitive task analysis (CTA), may not be sufficient to support identification of the detailed design specifications required for the development of an ITS for a complex training task incorporating multiple underlying skill components, as well as multi-modal information presentation, assessment, and feedback modalities. Our current work seeks to develop an ITS for training Robotic Assisted Laparoscopic Surgery (RALS), a complex task domain that requires a coordinated utilization of integrated cognitive, psychomotor, and perceptual skills.

**Results:**

In this paper, we describe a methodological extension to CTA, referred to as multi-modal task analysis (MMTA) that elicits and captures the nuances of integrated and isolated cognitive, psychomotor, and perceptual skill modalities as they apply to training and performing complex operational tasks. In the current case, we illustrate the application of the MMTA method described here to RALS training tasks. The products of the analysis are quantitatively summarized, and observations from a preliminary qualitative validation are reported.

**Conclusions:**

We find that iterative use of the described MMTA method leads to sufficiently complete and robust task models to support encoding of cognitive, psychomotor, and perceptual skills requisite to training and performance of complex skills within ITS task models.

## Background

The practice of task analysis (TA) is used in the design and development of procedures, training, and evaluations across a wide variety of human-performed as well as automated skills and tasks. While early TA studies often focused on relatively simple procedural and physical tasks, over the years, TA methods have been applied to increasingly complex tasks with increased emphasis on the cognitive components of such tasks; cognitive task analysis (CTA) typically refers to methods focused primarily on the cognitive processes associated with proficient or expert performance of operational tasks, including reasoning, problem-solving, and decision-making (for a detailed review, see Hoffman & Militello, 2012). Within the context of more complex tasks that were not purely behavioral in nature, CTAs particularly sought to capture the decisions and analyses that could not be directly observed, as well as the deeper conceptual knowledge that served as the basis for analytical strategies (Clark, Feldon, vanMerrienboer, Yates, & Early, 2008). CTAs have shown to be effectively applied to declarative knowledge, procedural knowledge, and decision points, as well as complex cognitive tasks incorporating multiple types of knowledge and processes (Clark et al., 2008).

The specific methods of task analysis, as well as their outcomes and applications, differ vastly depending on the objectives of the TA (Silber & Foshay, 2009). For example, a TA focused on the development of a training system may require not only characterization of the correct way of performing the targeted tasks, but also elucidation of optimal ways for teaching correct task performance, including identification of common mistakes, helpful hints and cues, and appropriate assessment and feedback techniques. Furthermore, the underlying knowledge, skills, and abilities/attitudes (KSAs) associated with a particular task or domain dictate the methods necessary to effectively decompose task characteristics. For example, procedural tasks involving ordered steps may require a hierarchical task analysis (HTA) approach (Annett, 2003).

Specifically in the field of training, a tradition of conducting task analysis exists, and in many cases CTA is used to develop curriculum content within the medical domain (e.g., Velmahos et al., 2004; Johnson et al., 2006). Behavioral task analysis (Jonassen et al., 1998) is used to determine what should be taught and what is the best way of training students. This way of thinking naturally leads to modeling how experts perform well-defined tasks (e.g. performing routine engine maintenance). Extensions of this practice to learning domains that not only exercise procedural knowledge but also conceptual knowledge are discussed within the field of cognitive task analysis (Clark et al., 2008), which retains the principle of modeling expert performance.

While some TA methods rely primarily on real-time “think aloud” protocols in which individuals explain what they are thinking and doing while completing a task, others involve post hoc assessments, including the use of video recordings to conduct “play-by-play” analysis of one’s own recorded task scenarios or by other individuals (e.g., Schlager, Means & Roth, 1990).

In our ongoing work, we are developing scalable training technologies that make expert human-level personalized instruction available to learners. We are extending intelligent tutoring technologies developed for conceptual domains such as high-school level Mathematics and Science to complex task domains that not only involve recall and application of conceptual knowledge, but also precise display of psychomotor skill and perceptual acuity. Specifically, in our current efforts, we are developing an ITS that will augment simulation based training currently used by surgeons learning to use a sophisticated robotic surgical system. Training surgeons to utilize robotic surgical devices such as the da Vinci Surgical System (DVSS) is a prime example of a complex task domain involving integrated cognitive, psychomotor, and perceptual skills; and for which a set of well-defined training tasks have been developed. With the increasing use of these surgical systems, reaching over 3 million cases worldwide to date (Intuitive Surgical, 2016), there is a dramatic increase in the need for effective training and assessment of the surgical skills required to perform medical procedures using these systems. Of particular importance is the initial learning curve associated with acquisition of these skills by inexperienced surgeons, which has numerous implications, particularly in terms of patient safety (Hopper et al., 2007). While prevailing training practices involve the use of sophisticated high-fidelity simulation based training as well as on-device training, instruction and feedback currently require expert surgeon supervision. Meeting this training need places an additional training burden on clinical surgical staff who are primarily engaged in catering to patients’ need for these surgical procedures.

In addition to incorporating traditional TA methods into the design and development of such training systems, empirical research involving objective measures is often helpful in identifying underlying characteristics of task performance and aspects of task performance that result in a large degree of variability across individual performers. For example, within laparoscopic tasks involving tissue pulling, Lamata et al. (2008) found a logarithmic relationship between perceptions of tissue consistency parameters by comparing subjective valorizations with objective parameters such as peak forces applied to tissues in vivo.

Developing the ability to perform surgical procedures requires mastering complexity in multiple dimensions. Requisite cognitive skills include declarative information recall, procedural knowledge, and decision-making, as well as skills and abilities relevant to communication and situation awareness such as working memory capacity. Perceptual skills integral to surgical performance include both visuo-spatial and perceptual-motor skills including; but not limited to, depth perception and discrimination, recognition, interpretation, and often transformation of visual information. In the case of laparoscopic surgery this includes transformation of 2-dimensional visual information while working within a 3-dimensional physical space (Stefanidis et al., 2006). Relevant perceptual-motor skills within laparoscopic surgery include haptic or tactile perception (Singapogu, 2012) and proprioception within a context in which a mismatch exists in visuo-motor mapping between the visual image and the hands (Cao et al., 1996). Mature attending surgeons must be competent in all of these skills to effectively lead a surgical team through a procedure in which a patient’s health and life are at risk. Accomplishing all of this has led to extensively long educational programs, which typically include a four-year bachelor degree, a four-year medical degree, a three to five year residency, and perhaps a two-year fellowship. At the same time, the explosion of medical tools and technologies has led to ever increasing numbers of available procedures and accompanying increase in specialized knowledge. Acquiring more knowledge and skills by increasing the number of practice hours is not possible. Although, in many countries, safety regulations have limited the number of hours that a resident or fellow can learn and practice during this process. One alternative is to improve the methods of training, ideally increasing the speed at which mastery can be attained through more efficient training methods.

Virtual reality (VR) training environments have been shown to be useful objective assessment tools for evaluating psychomotor skills for laparoscopic surgery (e.g., Gallagher, Richie, McClure, & McGuigan, 2001), in addition to providing effective means for training in such complex skills. VR training environments for laparoscopic surgery range from simplistic so-called box trainers using a cell phone or tablet to high fidelity procedural simulators with advanced motion tracking metrics. The Fundamentals of Laparoscopic Surgery™ (FLS) program, developed by the Society of American Gastrointestinal and Endoscopic Surgeons (SAGES), is currently the “gold standard” for training, assessment, and certification of laparoscopic surgery skills in the United States, as well as many other countries. This didactic training and assessment protocol consists of declarative knowledge training materials and an associated computer-based test, as well as training of five manual skills tasks to specified proficiency levels using a video trainer box. The use of the FLS manual skills tasks has been demonstrated to be a valid method for both teaching and assessing psychomotor skills in laparoscopic surgery based on randomized controlled studies (Westwood, Hoffman, Stredney, & Weghorst, 1998). Several studies have also demonstrated that virtual reality training translates to improved laparoscopic skills in the operating room. In particular, FLS box trainer scores have been shown to be independently predictive of intraoperative laparoscopic performance as measured by the Global Operative Assessment of Laparoscopic Skills (GOALS) (Fried et al., 2004; McCluney et al., 2007; Soper & Fried, 2008; Sroka et al., 2010). However, the FLS box trainer tasks are designed to assess psychomotor skills in isolation from the cognitive skills required to perform complex surgical procedures.

Intelligent Tutoring Systems (ITS) have been shown to be particularly valuable for teaching cognitive tasks such as troubleshooting, problem solving, and resolving critical situations. As a human tutor does, an ITS continually monitors and assesses the individual student's actions, infers the student's state of knowledge, and decides on the next instructional move to maximize the student's learning based on an embedded student model, task model, and instructional model. As highlighted by a recent meta-analysis (Kulik & Fletcher, 2016), research and development within the domain of ITS has demonstrated the technical feasibility and relative effectiveness of computer-based adaptive instruction as compared to classroom and small group instruction. ITS development has been applied across multiple domains, including within military applications such as ship handling and tactical decision-making. Furthermore, previous development efforts have demonstrated the ability to effectively apply generic ITS components such as authoring tools to specific military domains (Stottler et al., 2001; Sottilare & Holden, 2013).

Motivated by this training need and the feasibility of extending the envelope of existing technology, we are building an ITS for training surgeons to use robot-assisted surgical devices, specifically to use the DVSS system for high demand laparoscopic procedures. This motivating context informs the task analysis that is documented in this article. Specifically, we have conducted task analysis of a set of well-defined robotic surgery training tasks, developed and validated under the Fundamental of Robotic Surgery (FRS) curriculum, which are elaborated in the next section. We have developed a modified task analysis technique to be able to capture the complexity rising from the interaction of cognitive, psychomotor and perceptual skills, together referred to here as the multiple modalities of (or multi-modal) skills involved in this domain, and also to identify optimal instructional strategies, assessment metrics, and multi-modal feedback for guiding acquisition of these skills. Representative products of the multi-modal task analysis (MMTA) method presented here are based on considerations for inputs necessary to encode expert knowledge into the task model component of the ITS being built.

### Robot-assisted laparoscopic surgery (RALS)

Originating from the military’s vision for telemedicine, surgical robots were intended to provide patients (e.g. injured soldiers) in remote locations access to advanced medical care. In their present realization however, surgical robots are used to perform on-site complex procedures in which the surgeon is decoupled from the patient. Instead of controlling the surgical instruments by direct hand manipulation, surgeons use a console, which in turn tele-manipulates surgical instruments that are mounted on the arms of a robot. This type of robot-assisted surgery provides increased precision, flexibility and control to a surgeon compared to traditional practice. For example, the surgeon is in a seated position at the console, has the freedom to relax or reorient his/her hands without moving the surgical instruments and has better access to the surgical area. Additionally, compared to traditional laparoscopic surgical systems, robot-assisted laparoscopic surgery (RALS) offers stereoscopic vision that offers better depth perception for the surgeon.

A prominent surgical robot is the da Vinci Surgical System (DVSS) which is presently used to perform a variety of minimally invasive surgical procedures within the areas of gynecology, urology, neurosurgery and cardiology. While additional makes and models of surgical robots are also being commercially marketed, presently the DVSS is the most widely deployed globally and has been used to perform over 3 million surgical procedures.

### Current training regimen

RALS practice in its current state requires highly trained surgical staff. Specifically, the surgeon at the console requires significant training to safely and successfully complete procedures using the robot. In response to this need, training regimens have been developed and are being used across the spectrum of institutions engaged in training surgeons. However, a standardized curriculum for RALS training does not exist (Foote & Valea, 2016).

Training for using surgical robots is conducted in the form of courses. Surgeons typically receive instruction on the necessary psychomotor skills in isolation from the cognitive and perceptual skills, and may only perform these skills in an integrated manner during a single-day course on an animal model. Following an introductory orientation to robotic surgery, surgeons train under the mentorship of a practicing robotic surgeon at their respective hospitals. None of this guidance is standardized, validated, or accredited by outside professional groups, although efforts are underway to develop certification programs for robot-assisted surgery. The FRS, which we will describe in detail, represents a leading effort on developing multi-specialty surgical skill education, training, and assessment program, including standardized objective measures of RALS proficiency in psychomotor skills that have been determined to be fundamental across specialties.

Another key development that informs the current robot-assisted surgical training practice is the availability of simulation-based training systems that reduce the cost of training and provide access to an increasing collection of training content (simulations). We will also present an overview of simulation-based training for RALS later in this section.

In addition to the availability of a well-defined set of standardized training tasks within the FRS curriculum and high fidelity simulated FRS training scenarios within existing robotic surgery simulator platforms, this use case provides several additional advantages. Both existing and emergent training scenarios can be augmented with additional external pedagogical controllers with relative ease due to a collaborative relationship with an existing RALS simulator company; this makes a case for the feasibility of timely development of an ITS that would observe, support and assess trainees performing FRS tasks in the simulation. Furthermore, upon development and validation, this ITS would be potentially ready for use within prevailing medical training practice for training novice robotic surgeons as well as for refresher training and assessment for expert surgeons.

### Fundamentals of robotics surgery (FRS)

The FRS curriculum is a multi-specialty robotic surgical skills education, training, and assessment program that was developed through a series of consensus conferences involving expert surgeons, medical educators, behavioral psychologists, and cognitive scientists. The goal of the FRS development process was to develop a proficiency-based curriculum of basic technical skills that surgeons could be trained and assessed on to prepare them for performing robot-assisted surgical procedures across a wide range of specialties.

The FRS curriculum, in its current form, is divided into four modules, including an introduction to surgical robotic systems, didactic instructions, psychomotor skills tasks, and team training. Of specific interest to our current work is the psychomotor skills curriculum that is comprised of six training tasks. These training tasks have been implemented both as a physical training model suitable for use on the da Vinci surgical robot as well as simulation-based training available on multiple RALS simulator manufacturers. Table 1 lists these tasks along with brief descriptions and listing of surgical skills that each task is intended to exercise. A snapshot of each task being performed in a simulation based training environment is also shown.

**Table 1:**
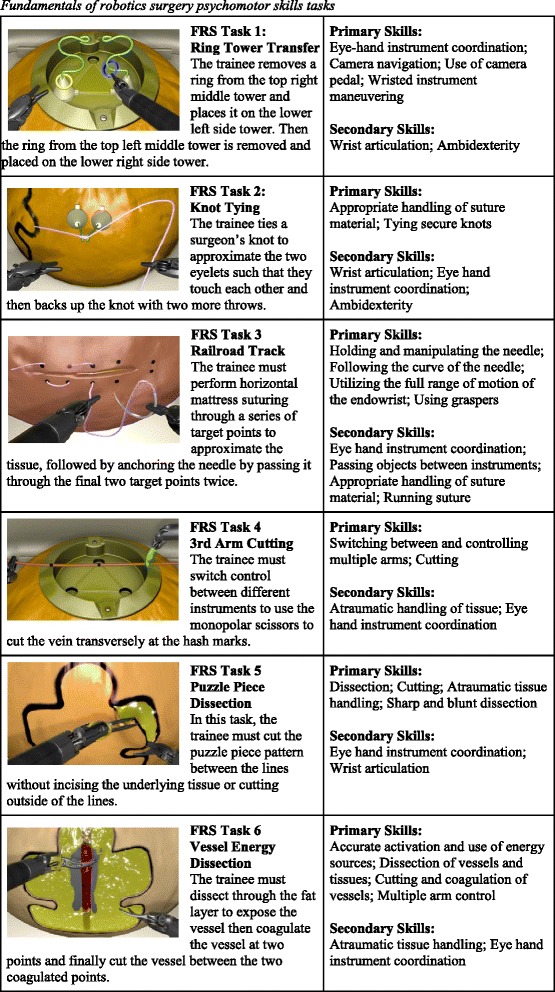
Fundamentals of robotics surgery psychomotor skills tasks

Despite the FRS terminology used to characterize these tasks, they exercise not only the psychomotor skills listed in Table 1 but also the cognitive and perceptual skills used in performing surgical procedures. While the task analysis described in this paper is applied only to the FRS tasks, the scope of the ITS development effort extends to supporting training for surgical procedures. Specifically, we are currently working on developing task models for prostatectomy using the method described in this work. These surgical procedures employ the cognitive skills such as knowledge of anatomy and biosignals to a greater extent.

### Simulation-based training for RALS

Our approach to the development of an ITS for RALS (RALS ITS) involves the use of a simulator for robotic surgery procedures. The ITS operates as a controller of the simulation. Commercial availability and deployment of simulation-based training systems for RALS informs and enables this approach.

Simulation-based surgical skills training has been demonstrated to be effective, and can produce learning curves similar to non-simulation based learning (e.g., Hernandez et al., 2004), indicating that simulation could provide a safe and structured context in which to ascend the initial phase of the learning curve without exposing patients to increased risk. Currently, four different simulation systems are available for training and developing skills in robotic surgery, each attempting to convey a standardized curriculum that can be assessed via standardized metrics, while providing limited assistive cueing and feedback to support a level of unsupervised learning (Smith et al., 2015). Three of the four simulators have demonstrated the ability to improve basic robotic skills, resulting in skills comparable with those obtained on dry laboratory simulation (Bric, Lumbard, Frelich, & Gould, 2016). Training, however, continues to rely heavily on instruction and guidance provided by a human instructor.

**Fig. 1:**
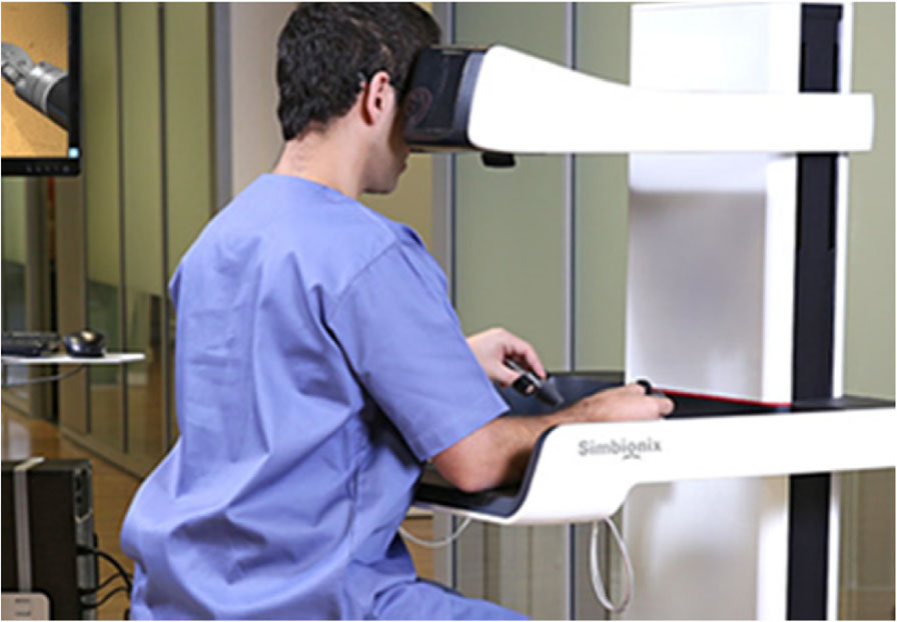
RobotiX Mentor Simulation System

In our current work, we are using the RobotiX Mentor simulation system developed and marketed by Simbionix because of the availability of large collection of curriculum content which includes simulated FRS tasks as well as several surgical procedures (Prostatectomy, Hysterectomy, Vaginal Cuff Closure and Lobectomy). Furthermore, the RobotiX Mentor offers a hardware implementation of the surgeon console that is close to the DVSS and accurately models the robot’s kinematics, tools and workspace. The RobotiX Mentor features a stereoscopic personal display and life-like graphical rendering of the FRS tasks as well as anatomical space. Finally, the choice of this simulation system for prototyping our ITS is informed by technical reasons including the off-the-shelf computing hardware used by this simulator and an ongoing effort to expose an application programming interface (API) that would allow the ITS to observe student behavior in real-time and control the simulation’s behavior in various ways necessary to deliver interactive support to the trainee. Figure 1 shows a photograph of the RobotiX Mentor. The FRS task snapshots shown in Table 1 were rendered by this simulator.

### Intelligent tutoring system for RALS

In our recent work, we have developed an online learning platform for creating and delivering problem-solving based learning tasks to students. In addition to the wide applicability of problem-solving as a learning activity in secondary and higher education, scalability goals are accomplished through domain independence and online access. The ITS embedded within this learning platform uses example-tracing (Aleven et al., 2009) based task models to represent optimal solutions to learning tasks (problems). Example-tracing is a technique used in intelligent tutoring systems to develop task models. A set of *example* demonstrations of ways of successfully completing a task are collected from individuals directly performing the task. These demonstrations are used to construct a behavior graph, which is a directed graph that models multiple ways to solve the problem. During tutoring, student’s steps are traced against this model i.e. the behavior graph to interpret the student’s actions. This example-tracing approach to tutor development requires less effort and expertise than other approaches such as model tracing tutors, in part due to well-developed general-purpose authoring tools such as the Cognitive Tutors Authoring Tools (CTAT) (Aleven et al., 2006) and the ASSISTment Builder (Razzaq et al., 2009). To further reduce the effort in constructing task models, we have developed tools and techniques for automating the development and maintenance of these task models using fine-grained log of behavior demonstration by multiple experts (Kumar et al., 2014).

One of the caveats of the above application of ITS technology arises from the relative simplicity of the learning tasks, and correspondingly task models, taught in secondary school subjects like mathematics and physics. Training domains such as RALS require an extension of the task modeling techniques employed in cognitive domains such as problem-solving in order to also model the skills exercised in perceptual and psychomotor domains within the underlying ITS framework. Furthermore, despite the effectiveness of ITS, not many fully developed applications of intelligent tutoring technologies have been developed for medical training. Some of the earlier tutors from the medical training domain included: medical diagnosis training (Clancey, 1983, 1986), teaching drawing conclusions from diagnostic reasoning (Voytovich, 1985), how to interpret mammograms (Azevedo & Lajoie, 1998), teaching diagnostic reasoning for antibody identification (Smith et al., 1998), teaching the interpretation of neuroradiological images (Sharples, et al., 2000), teaching how to detect diagnostic errors in internal medicine (Graber et al.,^2005^), teaching clinical medicine using various media (Martens, et al., 2001), teaching medical students to develop high level pedagogic strategies (Yudelson et al., 2008), and training pathologists using natural language (Saadawi, et al., 2008). More recent research and development in this domain has examined very specific use case applications such as gist comprehension and knowledge about genetic testing for breast cancer risk (Wolfe et al., 2015), as well as underlying processes within targeted use cases. For example, Duffy et al. (2015) examined the nature of cognitive, metacognitive, and affective processes among a medical teams managing challenging simulated medical emergencies. Duffy, Lajoie, and Lachapelle (2016) also explored the detection, tracing, and modeling of emotional processes during learning within medical education contexts. Furthermore, recent medical ITS development has increasingly focused on web-based platforms such as the Virtual Civilian Aeromedical Evacuation Sustainment Training (VCAEST) (Shubeck, Craig, & Hu, 2016). The still relatively limited application of ITS to medical training as well as the need to extend ITS component technologies (such as task models) to the complex learning tasks involved in medical training informs our development approach.

**Fig. 2:**
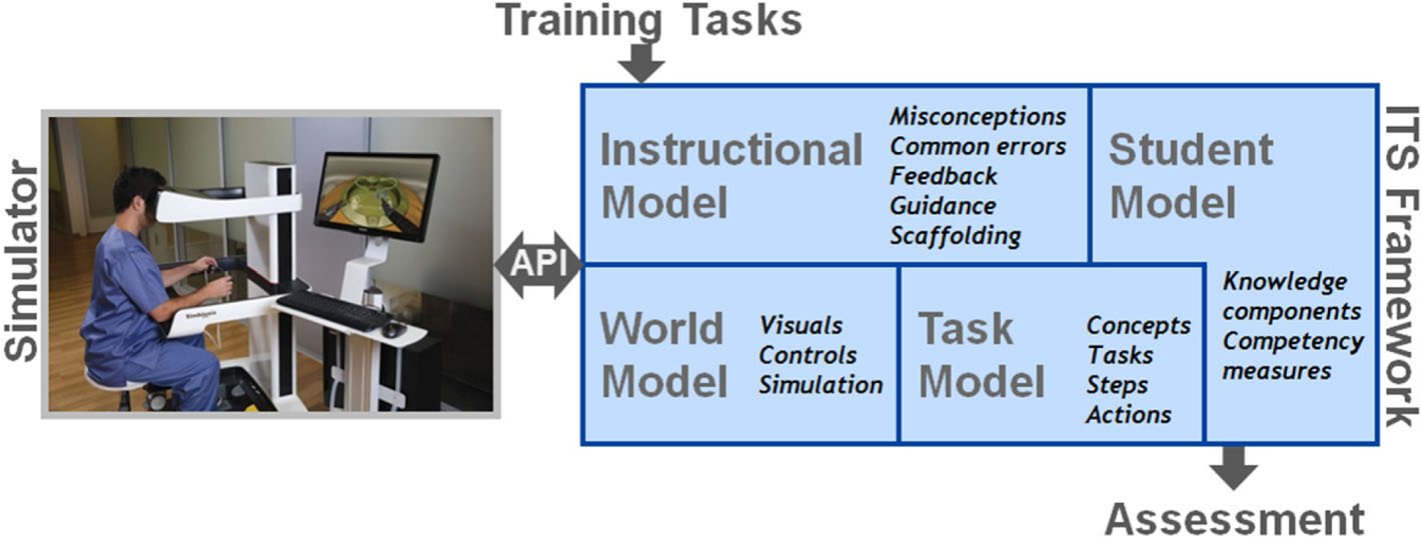
High-level design of RALS ITS

High-level design of the RALS ITS is shown in Fig. 2. A RALS simulation system is connected to the ITS framework through an integration API. As discussed in the previous section, we will use the RobotiX Mentor simulation system to develop the first prototype of the RALS ITS. The ITS is built upon a framework that includes state-of-the-art computational models of the learner skill proficiencies and task procedures. While this framework will be developed to be widely extensible to other training of medical procedures, in our current research and development efforts the framework will be customized for the RALS FRS tasks and surgical procedures included in the prototype (prostatectomy). The ITS observes a student’s performance of a surgical task within a simulated environment and, much like a human tutor, offers guidance to the student through four intelligent capabilities: (1) a model of the environment in which the task occurs, including knowledge of the simulator, its controls and visualizations, (2) an expert level understanding (or model) of the learning task being performed, (3) an ability to assess the student’s proficiencies at the various skills required in the tasks by observing the student and (4) the ability to apply pedagogical best practices to steer the student’s learning through an optimal path. The task model of the ITS is informed by the multi-modal task analysis (MMTA), which we describe in detail in Section 4. The student model statistically analyzes observations of the student’s performance against established performance standards to generate a profile of the student. Finally, the third capability is realized through the ITS instructional model.

An integration API specifies the interaction between the framework and the simulator to enable ITS access to student performance data collected within the simulator as well as to inject pedagogical interventions produced by the ITS. Our long-term goal is to make the ITS agnostic of the simulators that implement this API specification.

Given the inherent complexity of developing an ITS for a task domain such as RALS, a critical element of the design process is the task analysis, which must be capable of generating highly granular requirements and specifications to drive the development of effective instructional, world, task, and student models. In particular, as the resulting ITS must be capable of providing real-time guidance and feedback regarding the various underlying cognitive, perceptual, and psychomotor skills involved in performance of RALS tasks and procedures, it is necessary to explicitly decompose each of these contributing factors in isolation, but also to determine optimal methods for providing both isolated and integrated performance-based feedback. Furthermore, while a traditional ITS typically focuses on training primarily cognitive skills, integration of ITS capabilities within a RALS simulation environment requires detailed specifications regarding psychomotor and perceptual skills assessment and instruction in order to develop models that account for all aspects of the targeted tasks. In order to accomplish this, a need was identified to develop a novel TA method for application to a series of RALS tasks.

## Methods

### Multi-modal task analysis design

The objective of this effort was to leverage best practices and existing TA methods to develop a novel approach to complex task decomposition and analysis that would enable highly granular modeling of both isolated and integrated task components, as well as identification of metrics necessary to assess performance and provide intelligent instruction in real-time. For example, advanced hand motion tracking metrics can be used to provide granular assessments of psychomotor skills. The MMTA was specifically designed to develop simulation-based training for complex tasks involving cognitive, perceptual, and psychomotor skills. While there has been increasing emphasis on both technical and non-technical skills within the surgical domain, and development of multiple evaluation techniques, these skills are largely trained and assessed in isolation (Dedy, Bonrath, Zevin, & Grantcharov, 2013). As demonstrated by Skinner (2014), psychomotor and cognitive/perceptual skill relevant to laparoscopic surgery are acquired and decay at differential rates when performed in isolation as compared to performance of the same skills in an integrated manner.

Particularly in the case of a RALS simulation-based training environment, cues and feedback to the user could be in the form of a wide variety of visual, auditory, and even haptic stimuli. This required an emphasis on identifying current and potential metrics to determine how best to facilitate real-time root cause error analyses and appropriate error correction feedback. Therefore, the TA methods had to explicitly decompose the current task modality parameters and also identify potential novel multi-modal cues and feedback to be integrated within the ITS.

Multi-modal processes have been used in the past for training system design, but with an emphasis on cue fidelity for simulator design. For example, Milham et al. (2008) describe a Sensory Task Analysis (STA), which is used to identify multi-modal cues experienced during real-world performance and their associated functionalities. The primary goal of the STA is to gather rich contextual data, which are then leveraged in the training system design. For each task and subtask in the STA, multi-modal cues (visual, auditory, haptic, etc.) that trainees gather and act upon within the real world operational environment in order to successfully complete the task are identified. These multi-modal cues can then be replicated within a simulated environment at various levels of fidelity (Muller et al., 2006). The MMTA seeks to extract salient multi-modal cues from instances of real world task performance, but also for existing training systems. For example, the MMTA conducted for FRS tasks presented in this paper included detailed evaluations for the tasks performed on both the physical FRS dome using the DVSS robot as well as the simulated versions of the FRS tasks on the RobotiX Mentor.

### Previously established CTA technique

The MMTA method builds on an established CTA technique developed by Cannon-Bowers et al. (2013) for simultaneous generation of training requirements, performance metrics, scenario requirements, and simulator/simulation requirements for medical tasks using a scenario-based approach. This approach requires experts to perform targeted tasks multiple times, with each iteration probing a different dimension of the training development process, and includes the following steps:

Step 1: **Select subject matter experts (SMEs).** SMEs must be able to give detailed accounts of task/training requirements. Typically, multiple SMEs are selected. Instructors are often used as SMEs.
Step 2: **Elicit the major tasks and subtasks.** The major steps and sub-steps needed to accomplish the training task are collected by administering a “think aloud” protocol to the SMEs as they perform the training task. A human patient simulator maybe as used a reference point to help them recount how the task is performed.
Step 3: **Elicit critical cues.** The SMEs then perform the task again, and the task analyst(s) interview the SME using probe questions to elicit critical cues at each step. These usually include visual or tactile cues that serve as pre-conditions (i.e., if the step should be performed) or post-conditions (if it has succeeded). The cues are often documented along with applicable contexts, affordances and constraints.
Step 4: **Ask specific questions pertaining to deficiencies in simulation.** During this step, the SMEs are asked specific questions pertaining to deficiencies in prototype simulation and training devices after interacting with these devices. This step becomes increasingly prominent as the task analysis iterations progress alongside simulation development.
Step 5: **Elicit specific errors.** Lastly, task analysts elicit specific annotations about each level of the task decompositions that include common errors likely to be made by novices and specific trainee behaviors that typify each step.

This CTA approach provides an excellent model for a multi-step process targeting various aspects of simulation-based training design, which is broadly applicable to training systems design across a range of domains. The MMTA sought to expand on this method, including several key differentiating components. First, the MMTA adapts Step 3 of the above process to specifically target individual skill components (cognitive, perceptual, and psychomotor) and to emphasize the use of multi-modal information presentation via visual, auditory, and haptic stimuli to optimize intelligent instruction. Furthermore, the MMTA process and analysis was designed to not only capture the behaviors and observations of skilled practitioners and instructors, but also of novice^1^ and intermediate users to capture non-optimal and inaccurate deviations from the best path to performing a training task within the task model. Therefore, unlike the CTA method used by Cannon-Bowers et al. (2013), which included skilled practitioners and instructors, the MMTA design included surgical trainees with varying levels of experience within the RALS domain. Finally, the MMTA method, detailed in the following section, includes a step involving experts from a similar but distinct specialty domain in order to identify specialty-agnostic instructional design requirements and to assess generalizability of the resulting TA products.

### MMTA technique

The MMTA method includes specified approaches for capturing, analyzing, and representing ideal skills performance and common deviations from the ideal, as well as associated multi-modal cues, instructional strategies, feedback, and metrics. This method provides a novel approach to task analyses across complex training domains, and particularly for medical training tasks which exercise complex integrated skills. The perceptual and psychomotor aspects of medical training tasks make video recording a critical component of this approach to enable targeted probing and decomposition of underlying cognitive, psychomotor, and perceptual processes, which may be performed in an integrated manner, but require initial training at the component task level. This level of decomposition is critical from the perspective of ITS design as the ITS must have the ability to determine which underlying skill components are contributing to overall performance in order to provide appropriate tailored training content and support to the trainee.

This approach seeks to address a common challenge in knowledge elicitation from skilled experts as they often have reached a stage of automaticity in which multiple steps and underlying skill components are no longer differentiated at the conscious level; thereby making it difficult for experts to articulate low level processes and steps. Video recording and repeated replay of the targeted tasks during task decomposition enables the researcher 1) to pause the video at various points to probe the expert for finer level details, and 2) to emphasize specific task or skill subcomponents during each iteration of the video review process. For example, the researcher might first review the video with an expert, focusing only on cognitive task components, processes, and skills; then review the video a second time, focusing only on psychomotor components, and a third time focusing only on perceptual components.

The MMTA method includes the following steps, which could be applied across a variety of task domains:

Step 1: **Review relevant materials.** Materials used in this step may include documentation, texts, videos, and existing training content relevant to the tasks in the form of available formalized curriculum, texts, and publications. In the case of some tasks and domains, this may also include technical manuals for equipment and tools involved in task performance. In some cases the later phases of the MMTA may reveal inconsistencies between formal documentation and standard practice within a domain. Identification of such inconsistencies may serve to elucidate best practices that have been established through practice and perhaps handed down via formal and informal apprenticeship and training models, but not formally documented.
Step 2: **Select subject matter experts (SMEs) and novices.** Domain experts and instructors with expertise in performing and/or training others to perform the tasks being analyzed are selected to participate as SMEs. Also, a range of novices are recruited to provide video recordings of task performance for subsequent SME interviews. Novices also participate as trainees to elicit real-time assessment, instruction, and feedback. Instructors are used as a particular type of SME as they are often able to better articulate cues used to determine if a trainee is performing well or poorly. They are also able to provide effective instructional strategies, which can be instantiated within the ITS.
Step 3: **Video record training and conduct structured interviews.** Novices are observed and video recorded as they perform the tasks while receiving real-time assessment, guidance, and feedback from an instructor. Targeted questions are asked of both the novice trainee and the instructor during and after task performance. The recorded performance is then reviewed with the instructor to determine whether existing assessment metrics are suitable, and to identify additional metrics that have the potential to improve real-time and post-hoc assessment. Instructional strategies, guidance, and feedback employed by the instructor are also explored, including tips and tricks that the instructor finds to be particularly effective. Instructors are also encouraged to think about ways in which multi-modal training technologies could be used to facilitate instruction and convey complex concepts within the targeted domain. The videos are used to assist in further task decomposition and brainstorming related to development of additional instructional approaches to improve training.
Step 4: **Video record instructors performing the task using a think-aloud protocol.** Researchers observe and video record a think aloud protocol for an instructor completing the relevant tasks, explaining how they are completing the task as if they were demonstrating proper task performance and performance strategies to a trainee. The emphasis of this protocol is on decomposition of proper task performance, rather than on demonstrating ideal task performance at a faster pace. The instructor is encouraged to pause as needed to demonstrate complex task components and may even demonstrate or describe common errors and root causes of those errors. The researcher likewise may ask the instructor to pause or slow down, and may ask targeted questions to support task decomposition, understanding of assessment metrics and techniques, common errors, and effective “tricks” or strategies for successful task completion. Following the think aloud task performance, the researcher may review the video with the instructor for further clarification to highlight underlying cognitive, psychomotor, and perceptual skills and instructor-specific feedback.
Step 5: **Video record novice task performance.** Domain novices are video recorded while performing selected tasks, primarily in order to generate videos for subsequent review by experts. However, this step in the process may serve several purposes. A think aloud protocol may be completed by the novice during task performance. The video could also be reviewed with a researcher following task performance, providing an opportunity for probing questions to determine novice strategies and perceived areas of difficulty. An expert or instructor could also provide real-time instructional guidance and feedback during novice task performance as well.
Step 6: **Review video with instructors.** Researchers review the novice videos with an instructor, probing to elicit underlying cognitive, psychomotor, and perceptual skill components, as well as skill integration in the form of knowledge concepts that can be applied to the student model. Videos are iteratively reviewed with probe questions designed to target underlying cognitive, psychomotor, and perceptual skill components, as well as skill integration in the form of knowledge concepts that can be applied to the training system student model. Probe questions can also be designed to target training requirements, performance metrics, and scenario requirements to be applied to the resulting system design.
Step 7: **Review video with domain-specific experts.** Multiple domain experts view the novice videos with a member of the research team asking probe questions to elicit critical multi-modal assessment cues, diagnose errors, and discuss potential training requirements and strategies for improved performance.
Step 8: **Review video with experts from a different specialty or domain.** One or more experts from a different specialty or domain view the novice procedure videos with a member of the research team asking probe questions to elicit critical multi-modal assessment cues, diagnose errors, and discuss similarities and differences across domains.

The following section provides details regarding the application of this process to the targeted RALS use case. Future work will seek to apply this same methodology to additional use cases and domains in order to assess generalizability.

### MMTA application to RALS

We evaluated existing robotic surgery skills training, primarily focusing on the FRS curriculum, which is designed to train and assess surgeons in the technical skills required to safely and efficiently perform robotic-assisted surgery (Smith et al., 2014), and conducted an MMTA for the six FRS tasks illustrated in Figure 2.

This data collection effort focused on the following steps in the MMTA method:

Step 1: **Review relevant materials**. In our case this included documents such as the Report from the FRS Consensus Conference on Outcomes Measures, training curriculum from the FRS online curriculum (frsurgery.org), promotional literature from robotic simulator companies, YouTube videos of RALS procedures and FRS tasks, and simulator training such as the RobotiX Mentor Simulator’s FRS skill module.
Step 2: **Select subject matter experts (SMEs) and novices.** RALS instructors were recruited from Florida Hospital Nicholson Center (FHNC), who currently conduct RALS training courses at the center. Expert surgeons were recruited from attendees at the 2015 American Association for Gynecologic Laparoscopists (AAGL) Global Congress on Minimally Invasive Gynecology, as well as from available expert RALS surgeons at FHNC. Four of those surgeons had performed over 1,000 RALS procedures. Novice RALS surgeons were also recruited from FHNC. Importantly, the novice RALS surgeons were specifically selected based on their relative expertise in conventional (non-robotic) laparoscopic surgery. This particular demographic of surgeons was selected based on the assumption that many surgeons seeking RALS training are likely to be experienced, or even expert, surgeons having originally trained and practiced laparoscopy. As RALS prevalence and the availability of robots continues to increase, many laparoscopic surgeons are seeking RALS training. These “novice” participants had in depth prior knowledge of general surgical principles, skills, and laparoscopic surgical equipment, as well as detailed knowledge regarding the targeted (or similar) tasks. For example, these participants may have had extensive experience in performing a laparoscopic hysterectomy or experience performing laparoscopic inanimate skills labs using tools similar to the FRS dome. In most cases these participants also had cursory exposure to the DVSS and/or RALS simulators, and therefore had some familiarity and comfortability with the interface, including camera and clutch usage. In addition to the novice RALS surgeons, additional participants were selected to represent true novices in the classical/traditional sense: having limited prior exposure to any aspects of the targeted tasks, including little to no understanding of general surgical principles or familiarity with relevant anatomy or surgical tools.
Step 3: **Video record training and conduct structured interviews**. All novices were asked to perform one or more of the targeted MMTA tasks, including FRS tasks and simulated partial surgical procedure tasks (i.e., vaginal cuff closure and hysterectomy). During these sessions, RALS instructors provided real-time assessment, guidance, and feedback (positive and negative) to the novices as if they were a trainee in a RALS course of instruction. One or more members of the research team took notes and asked targeted questions of both the trainees and instructors during and after each session. Immediately following performance of each task, the instructors reviewed the resulting simulator scores with researchers, highlighting aspects of task performance that are automatically captured by the simulator. The videos from these sessions were also used to assist in formal task decomposition and additional brainstorming to identify additional metrics, instructional strategies, guidance, and feedback to potentially improve training within the RALS ITS.
Step 4: **Video record instructors performing the task using a think-aloud protocol.** Within this step, instructors were asked to complete each of the targeted FRS tasks, while providing think aloud commentary to describe their actions and thought processes particularly regarding task difficulty and approach/strategy. A researcher recorded video and notes during these sessions and asked targeted questions before and after each task was completed to support task decomposition, understanding of assessment metrics and techniques, common errors, and effective “tricks” or strategies for successful task completion.
Step 5: **Video record novice task performance**. The research team identified optimal FRS tasks and surgical procedure modules based on currently available training modules, access to novices (and experts to review the videos) in the same specialty, generalizability, and applicability to the military. The novices recorded included an expert laparoscopist with limited robotic surgical experience and limited FRS exposure. Novices were recorded performing all six FRS tasks, as well as other RALS procedures, including a vaginal cuff closure.
Step 6: **Review video with instructors**. A member of the research team reviewed each novice task performance video with one or more RALS instructors, pausing the videos as necessary to allow the instructor to make or elucidate on a point, or to allow the researcher to ask targeted questions. In support of the task decomposition, probe questions were used at each step in the task in order to target cognitive, psychomotor, or perceptual subtasks and subskills; relevant training requirements; key performance assessment cues and metrics; critical multi-modal cues and feedback needed to assist the trainee; tasks components requiring skill integration in the form of knowledge concepts, and scenario requirements to be applied to the RALS ITS.
Step 7: **Review videos with expert surgeons**. The expert observing the novice in real-time was interviewed first; the expert reviewed the video, with his/her commentary audible, pausing as needed to enable the expert to make or elucidate on a point, explain his or her commentary (including technical jargon), or to allow the researcher to ask targeted questions about what the surgeon was cueing on when making assessments of performance and providing specific feedback. The same video was then viewed by additional experts without the commentary of the first expert audible, again pausing as needed to enable the expert to make or elucidate on a point, explain his or her commentary, or to allow the researcher to ask targeted questions. Lastly, the video was watched a second time by some experts, but with the commentary of the real-time expert audible. This enabled other experts to make observations regarding the real-time instruction provided, and in some cases provided an explanation for the approach the novice was taking. This also helped the researchers to determine what types of feedback were based on personal preference or surgical style, as opposed to specific safety and efficacy concerns.
Step 8: **Review video with expert surgeons from a different specialty.** Due to resource constraints, we did not review videos with expert surgeons from different specialties. Instead, as part of the MMTA validation process, we reviewed the MMTA products with a variety of FRS and RALS experts from different specialties including an expert pediatric RALS surgeon, and an expert military RALS surgeon.

## Results and discussion

### Products

ITS task models make it possible to compare the trainee’s task performance in the simulator against that of experts. The models must encode experts’ knowledge of the procedures, techniques, checks and other nuances of performing the training tasks that are collected and documented through this task analysis. Hence, the computational representation of the task model informs the products of the MMTA.

We will use behavior graphs as a task model representation. Behavior graphs have been widely used by ITS because of the robust representation and readability they offer. Behavior graphs are directed graphs. Nodes in behavior graphs correspond to valid task states. Edges represent behaviors that may cause progression (or regression) through task states. As a trainee performs the training task, a path is traced through these nodes by following the traversed edges. The path provides an interpretation of the observed behaviors of the trainee. Behavior graphs support multiple paths to completing a task. Elements of the graphs may be annotated with nuances that help the interpretation of trainee behavior or drive the pedagogical strategies of the ITS. Some of the annotations commonly used include skills associations, identification of common errors and feedback prompts. Authoring tools are often used to construct, annotate and maintain behavior graphs. For complex tasks, multiple interlinked behavior graphs can be used. Each graph may represent various levels of task decomposition hierarchy or behaviors corresponding to loosely coupled yet coordinated skills that must be exercised to perform the task.

In order to construct these behavior graphs, the task analysis method described above must produce (1) task flow diagrams that capture the task procedure and (2) task decomposition tables that document annotatable nuances.

### Task flow diagrams

Tasks flows are directed graphs that represent a hierarchical decomposition of the task into steps and substeps. Multiple levels may be used until the task is decomposed down to a finite vocabulary of atomic actions. This type of decomposition follows commonly used HTA task decomposition methods such as those described by Annett (2003). The atomic action vocabulary is incrementally developed and iteratively refined during the task analysis. In addition to providing sufficient granularity to represent the task flow, the list of atomic actions is informed by ITS implementation considerations. Atomic actions form conditional clauses that the behavior graph edges encode. In order to automatically trace behavior graphs, it is necessary that these atomic actions are detectable, by the simulator, when they are performed by the trainee.

**Fig. 3:**
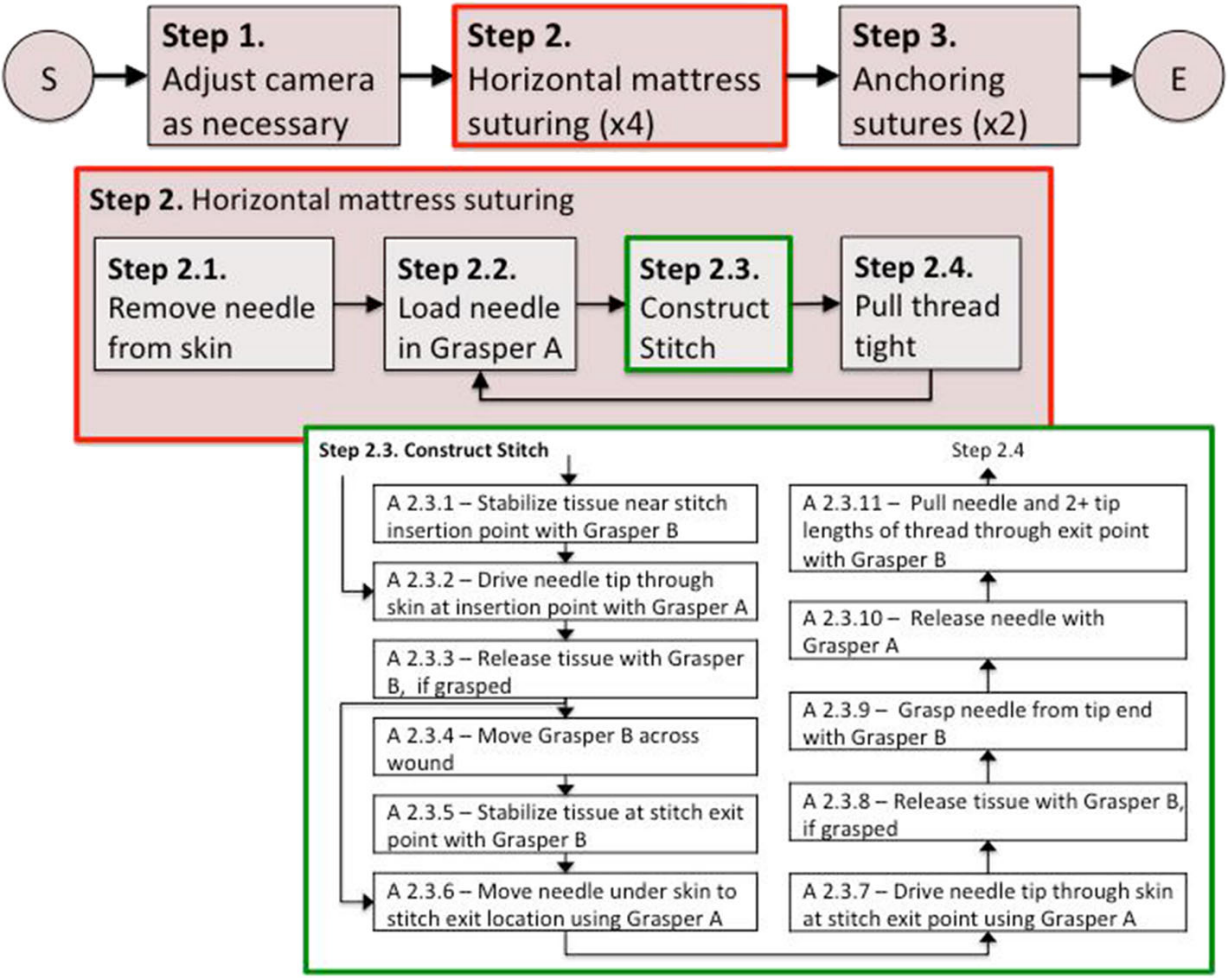
Task-flow diagram of a sub-step of an FRS task

The first iteration of the set of atomic actions used for encoding the FRS task flows was developed at the conclusion of the first iteration of the task analysis for these tasks. This set of 19 actions consist of the extracted and consolidated verbs such as cutting, grasping, moving an instrument, or pressing a pedal to activate surgical instruments or robotic limbs. Together with their related subjects and direct objects, they form a rudimentary vocabulary of describing the procedures of performing FRS tasks. Figure 3 shows an example of part of task flow diagram corresponding to the sub-step of forming a suture loop in FRS task 3. At the lowest level i.e. within step 2.3, the sub-steps represent atomic actions and are described using the atomic action verbs like stabilize, drive, release, move, pull and grasp.

### Task decomposition tables

While the task flows include task procedures that correspond to necessary steps and sub-steps as well as steps that may be taken under certain conditions, they do not document additional information collected during the task analysis that is encoded into the task models. This includes information like optimal paths, skills required in performing each step, instructional strategies (e.g. cues & feedbacks) and effected assessment metrics.

The task decomposition tables include a textual description of each of the multiple levels of task flow steps as well as a mapping from these steps to the surgical skills targeted/assessed, metrics, cues, optimal and sub-optimal strategies, and common/critical errors. Where applicable, mappings to standardized assessment metrics such as Global Evaluative Assessment of Robotic Skills (GEARS) metrics (Goh et al., 2012), and Robotic Objective Structured Assessment of Technical Skills (R-OSATS) metrics, are included. To illustrate, Table 2 lists the additional information document through the task analysis for one of the sub-steps shown in Figure 3.

**Table 2:** Information captured in task decomposition table for sub-step A.2.3.6

Description	Move needle under skin to stitch exit location using grasper A
Instructional Goals	1. Optimal needle orientation and motion 2. Optimal needle depth
CommonErrors	1. Instrument collision 2. Drive needle too deep into tissue
OptimalStrategy	Bring needle tip in contact with the under side of the second target point by supinating hand A wrist slightly (if initial target is on top) or pronating hand A wrist slightly (if initial target is on bottom), maintaining perpendicular orientation and without driving needle too deep into the tissue
Non-optimal Strategies	1. Align needle tip with the under side without supinating dominant hand. 2. Align needle tip without maintaining perpendicular orientation.
Metrics	1. Number of instrument-instrument collisions 2. Time and economy metric 3. Number of instrument movements 4. Instrument path length 5. Number of unnecessary needle punctures 6. Hand orientation 7. Needle driving depth

While the products resulting from the above application of MMTA are too voluminous to be included in this document, Table 3 presents quantitative measures of the flow diagrams resulting from the task analysis of six FRS tasks. All of the FRS tasks were decomposed into three levels (steps, sub-steps and actions) as illustrated in Figure 3. We noticed that while some of the atomic actions (move, press, release and grasp) are used across all the tasks, the other atomic actions have specialized use only in a fraction of the FRS tasks. For example, drive as an atomic action is only used in the railroad track task which is the only task that involves driving a needle. We asked two robotic surgery instructors to rank the six task in terms of relative difficulty, and found that the variety of actions used within each task had the highest rank correlation among quantitative measures of flow diagram complexity, with increased number of actions correlating to higher perceived complexity. FRS task 3 (railroad track) task also has the highest amount of iteration of a sequence of actions because the stitching sub-steps have to be repeated several times.

**Table 3:** Quantitative characteristics of FRS task flow diagrams

Task	#Steps	#Sub-steps	#Actions	Action Variety	Actions in loops
FRS Task 1	10	10	76	4	24%
FRS Task 2	4	13	85	6	20%
FRS Task 3	3	10	55	8	51%
FRS Task 4	7	7	35	7	20%
FRS Task 5	5	5	29	6	38%
FRS Task 6	5	10	40	7	28%

### Validation

The MMTA task flows and task decomposition tables were validated by personnel from FHNC. The validation procedure starts with a surgeon performing the each of the FRS task on the simulator. The surgeon’s task performance is directed by reading out the task flow sequence captured in the MMTA products. As the surgeon performs these tasks, any occurrence of errors, lack of clarity in the documented steps, necessary deviations are identified and documented. In our validation of the FRS task flows, we found that the surgeons were able to successfully complete the tasks while following the read-out task flows.

Furthermore, the products resulting from the MMTA is compared with documentation of FRS task procedures and inconsistencies are noted. This is also applied to available documentation of metrics for each task. Specific inconsistencies that were found during this step of the validation pertained to differences in terminology used in medical literature, especially across specialties. For example, identical maneuvers to tighten a suture were named differently by different groups of surgeons (e.g., pulley maneuver vs. thread sweep).

**Fig. 4:**
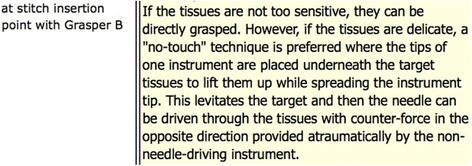
Alternative technique suggested by an expert surgeon during MMTA validation

The MMTA products for FRS tasks were reviewed by two expert surgeons who were previously not engaged in this task analysis. Both of these surgeons practice robotic surgery in different specializations. Both of these surgeons suggested modifications to the task flows and specific alternate techniques used in their specializations. For example, Fig. 4 shows a comment from one of these surgeons who suggested an alternative technique that can be used for stabilizing tissue which is more commonly used in pediatric practice.

When necessary, the tasks are repeated on a DVSS system (either on the FRS tasks simulation available on the DVSS or using the physical model) to identify and correct inconsistencies in the MMTA products arising from differences in fidelity and function of the RobotiX Mentor’s controls from the DVSS. As a last step of the validation, the issues identified are corrected through discussed among the task analysts and SMEs.

## Conclusions

This article summarizes the development of a novel approach to knowledge elicitation within the context of complex tasks for the development of highly granular TA products capable of driving design requirements for ITS models, and in particular for an ITS that is capable of targeting not only cognitive skills, but also integrated perceptual and psychomotor skills. This requires the development of complex ITS task, world, instructional, and student models, as well as the identification of appropriate metrics and algorithms to drive real-time performance assessment, error diagnosis, and intelligent feedback across these skill components in real-time. Furthermore, while a traditional ITS may use multi-modal cues and feedback to support cognitive skills training, the inclusion of psychomotor and perceptual skills components necessitates a deeper exploration of the ways in which visual, auditory, and even haptic modalities can be used to provide intuitive instruction. This required exploration of current methods of training by human instructors, as well as exploration of novel ways in which software-based instruction could emulate human instruction, or even provide alternative instructional strategies beyond what a human instructor is capable of providing.

The MMTA method leverages best practices in TA methods, including strategies for targeting the decomposition of complex tasks having underlying cognitive, psychomotor, and perceptual skill components, which must be performed in concert. Such tasks are challenging to perform, and perhaps more challenging to train as skilled experts and instructors may be unable to articulate the intricacies of optimal task performance or assessment.

The MMTA method, described here as a generalizable approach and also applied to a specific target domain, RALS, uses knowledge elicitation techniques involving both novice and expert robotic surgeons as they perform a series of procedural exercises. The process of examining the task from multiple perspectives, including novices, instructors, and expert surgeons, as well as iteratively extending and refining the MMTA products was a central characteristic of this MMTA process.

Results of the application of this method to the psychomotor skills curriculum of FRS and sample surgical procedure subtasks, which were reviewed and validated by expert robotic surgeons, will be implemented within a prototype RALS ITS. Through the validation process, we found that the task flows developed in this effort were complete and robust as demonstrated by a surgeon’s ability to perform these tasks while being read out the task flows; however, the current results are primarily qualitative. As we continue with the development of a RALS ITS and operationalize the task flows and decomposition tables produced in this work into task models, we will be able further validate their completeness as well as make necessary refinements through iterative application of pertinent MMTA steps. Furthermore, the resulting prototype ITS will be validated via a formal training effectiveness evaluation. Future work could also further explore alternatives to the MMTA method, comparing the resulting products to those resulting from more traditional TA methods.

More broadly, we posit that this method can be used for additional complex training domains that require cognitive, perceptual and psychomotor skills and knowledge such as maintenance tasks. It may be applied for the development of ITS task models specifically as well as for conducting task analyses in general. Future work will seek to apply this same methodology to additional use cases and domains in order to assess generalizability. Within the course of our efforts, we will be applying this method to additional surgical procedures (specifically prostatectomy).

## Abbreviations

AAGL: American Association of Gynecologic Laparoscopists
API: Application Programming Interface
CTA: Cognitive Task Analysis
DVSS: da Vinci Surgical System
FHNC: Florida Hospital Nicholson Center
FRS: Fundamentals of Robotic Surgery
GEARS: Global Evaluative Assessment of Robotic Skills
ITS: Intelligent Tutoring System
LS: Laparoscopic Surgery
MMTA: Multi-modal Task Analysis
RALS: Robot-assisted Laparoscopic Surgery
R-OSATS: Robotic Objective Structured Assessment of Technical Skills
SME: Subject Matter Expert
STA: Sensory Task Analysis

## Footnotes

1 While we use the term ‘novice’, these individuals were typically accomplished surgeons, but with limited or no prior exposure to robotic surgery.
